# Per-Oxide of Hydrogen (H^2^ O_2_)

**Published:** 1883-03

**Authors:** A. W. Harlan

**Affiliations:** Chicago


ARTICLE III.

### PER-OXIDE OF HYDROGEN ($H_2O_2$).

BY A. W. HARLAN, D.D.S., CHICAGO.

The editorial on Carbolic Acid in the January number of the INDEPENDENT PRACTITIONER was a pleasant article to read, because so few of our dental journals are in the habit of publishing practical articles concerning remedies in use by dentists, and it goes without saying that such articles are greatly needed by the majority of practitioners, who are too apt to fall into the habit of using remedies without investigating their claims to utility or effectiveness for the end desired, which may be to antisept, to destroy, to alleviate pain, or to promote absorbtion, all of which it would be difficult to accomplish with one remedy. But such is the force of habit that many of our fellow-practitioners will still continue to use carbolic acid for the multifarious purposes catalogued in the article referred to. It is not so much for the purpose of commending the editorial that I write now, as it is to call attention anew to per-oxide of hydrogen as an agent for the treatment of abscessed teeth, and particularly for the treatment of blind abscesses. I have had an experience with it in almost daily use for a year and a half, and am better satisfied with it for the above purpose than any other remedy that I have ever used.

If the reader chances to have a case in hand where he does not wish to cut through the process and there is a discharge through the pulp cavity, if he will cleanse the canal and then apply a pellet of cotton saturated with Fromsdorff's per-oxide of hydrogen, using no force, he will immediately see the liberation of oxygen which will combine with the pus or watery discharge, thinning it, until there will be a complete drainage of the sac beyond the apical foramen; then, as oxygen in its nascent condidion is an antiseptic, he will understand that it has been brought in contact with the walls of the sac, which is just what is demanded. Per-oxide of hydrogen is not an escharotic or an irritant. I use the volatile extract of eucalyptus as a dressing for the canal, inserting a shred of cotton fiber moistened with it, and stop the canal with a pellet of cotton dipped in dissolved gutta percha. I do not seal the cavity tightly, because I wish to leave a vacuum, in case I have not thoroughly applied the per-oxide. In three days the dressing is to be removed, and if there be any discharge from the sac the treatment must be repeated; if there should be no discharge, which is frequently the case, fill the canal with shreds of cotton dipped in the eucalyptus, and seal the cavity of decay with gutta percha firmly. After a week or ten days have elapsed the pulp canal may be filled in the usual manner. Tne rubber dam is to be applied to the teeth during the whole series of dressings; no saliva, alcohol, carbolic acid, or other medicament is to be used within the canal or permitted to have access to it. The per-oxide may be injected through the pulp canal if there is a fistulous outlet, and the apex of the root filled at once, and if there is no carious or necrosed bone around the point of the root, the abscess will heal in a few days.

Per-oxide of hydrogen should be protected from the light, and kept in a glass stoppered bottle in a dark corner. I have used small quantities from several manufacturers, and have found none so satisfactory as H. Fromsdorff's, manufactured in Erfurt, Germany. It is a valuable remedy applied locally in moquet ostitis, and for injectiom into the antrum. When an antiseptic is called for, I have made use of it in all the above affections with excellent results.

---

Article IV.

